# Sensor-Derived Measures of Motor and Cognitive Functions in People With Multiple Sclerosis Using Unsupervised Smartphone-Based Assessments: Proof-of-Concept Study

**DOI:** 10.2196/60673

**Published:** 2024-11-08

**Authors:** Matthew Scaramozza, Aurélie Ruet, Patrizia A Chiesa, Laïtissia Ahamada, Emmanuel Bartholomé, Loïc Carment, Julie Charre-Morin, Gautier Cosne, Léa Diouf, Christine C Guo, Adrien Juraver, Christoph M Kanzler, Angelos Karatsidis, Claudia Mazzà, Joaquin Penalver-Andres, Marta Ruiz, Aurore Saubusse, Gabrielle Simoneau, Alf Scotland, Zhaonan Sun, Minao Tang, Johan van Beek, Lauren Zajac, Shibeshih Belachew, Bruno Brochet, Nolan Campbell

**Affiliations:** 1 Biogen Cambridge, MA United States; 2 Department of Neurology CHU de Bordeaux Bordeaux France; 3 U1215 INSERM University of Bordeaux Bordeaux France; 4 Ad Scientiam Paris France; 5 Biogen Paris France

**Keywords:** multiple sclerosis, sensor-derived measure, smartphone, cognitive function, motor function, digital biomarkers, mobile phone

## Abstract

**Background:**

Smartphones and wearables are revolutionizing the assessment of cognitive and motor function in neurological disorders, allowing for objective, frequent, and remote data collection. However, these assessments typically provide a plethora of sensor-derived measures (SDMs), and selecting the most suitable measure for a given context of use is a challenging, often overlooked problem.

**Objective:**

This analysis aims to develop and apply an SDM selection framework, including automated data quality checks and the evaluation of statistical properties, to identify robust SDMs that describe the cognitive and motor function of people with multiple sclerosis (MS).

**Methods:**

The proposed framework was applied to data from a cross-sectional study involving 85 people with MS and 68 healthy participants who underwent in-clinic supervised and remote unsupervised smartphone-based assessments. The assessment provided high-quality recordings from cognitive, manual dexterity, and mobility tests, from which 47 SDMs, based on established literature, were extracted using previously developed and publicly available algorithms. These SDMs were first separately and then jointly screened for bias and normality by 2 expert assessors. Selected SDMs were then analyzed to establish their reliability, using an intraclass correlation coefficient and minimal detectable change at 95% CI. The convergence of selected SDMs with in-clinic MS functional measures and patient-reported outcomes was also evaluated.

**Results:**

A total of 16 (34%) of the 47 SDMs passed the selection framework. All selected SDMs demonstrated moderate-to-good reliability in remote settings (intraclass correlation coefficient 0.5-0.85; minimal detectable change at 95% CI 19%-35%). Selected SDMs extracted from the smartphone-based cognitive test demonstrated good-to-excellent correlation (Spearman correlation coefficient, |ρ|>0.75) with the in-clinic Symbol Digit Modalities Test and fair correlation with Expanded Disability Status Scale (EDSS) scores (0.25≤|ρ|<0.5). SDMs extracted from the manual dexterity tests showed either fair correlation (0.25≤|ρ|<0.5) or were not correlated (|ρ|<0.25) with the in-clinic 9-hole peg test and EDSS scores. Most selected SDMs from mobility tests showed fair correlation with the in-clinic timed 25-foot walk test and fair to moderate-to-good correlation (0.5<|ρ|≤0.75) with EDSS scores. SDM correlations with relevant patient-reported outcomes varied by functional domain, ranging from not correlated (cognitive test SDMs) to good-to-excellent correlation (|ρ|>0.75) for mobility test SDMs. Overall, correlations were similar when smartphone-based tests were performed in a clinic or remotely.

**Conclusions:**

Reported results highlight that smartphone-based assessments are suitable tools to remotely obtain high-quality SDMs of cognitive and motor function in people with MS. The presented SDM selection framework promises to increase the interpretability and standardization of smartphone-based SDMs in people with MS, paving the way for their future use in interventional trials.

## Introduction

### Background

Accurate measurement of meaningful change in neurological function, essential for optimal patient management and development of new interventions, remains a significant challenge. This challenge is particularly salient in the context of clinical trials, in which infrequent, in-clinic assessments are often used as primary end points [1-3]. Such infrequent, and at times subjective [4,5], assessments may not capture typical function or subtle disease progression because complex neurological diseases often exhibit significant fluctuations within individuals and are heterogeneous between individuals.

The need for better assessment tools is particularly compelling in the setting of disease monitoring for multiple sclerosis (MS), where there is an unmet need for enhanced performance-based measures of function across motor and cognitive domains commonly impacted by disease progression. MS is a heterogeneous disease that often leads to impairment in neurological domains that extend beyond lower limb function, as assessed by walking distance range, including cognition, gait and posture, and fine motor function [1]. Monitoring of MS-associated disability progression is essential for patient management and development of MS treatments; however, it is complicated by the presence of relapses, fluctuation of symptoms due to illness or fatigue, and infrequent in-clinic assessment of function. The use of conventional clinical instruments, which rely on intermittent in-clinic, rater-dependent outcome measures [6,7], further adds to the complexity of measuring function and treatment effects. Assessment tools yielding more continuous outcome measures in free-living environments may improve measurements of the efficacy of new treatments across key functional domains affected by MS disease by providing more accurate and reliable measures. Consumer adoption of smartphones and wearable technologies has offered us the opportunity to move measurements from the clinic to remote unsupervised settings, with the potential for more accurate, sensitive, and objective performance measures of function. Greater accuracy and sensitivity are expected to result from reduced variability due to increased frequency of measurement [8-10]. Greater accuracy and sensitivity may also result from the ability to measure unique aspects of function not currently captured by conventional clinical assessments [11]. This principle has been demonstrated by the fact that more people with MS show confirmed disability worsening when assessing change across multiple functional domains relative to assessment [12-15] based on the Expanded Disability Status Scale (EDSS) [16] alone. Sensor-derived measures (SDMs) extracted from digital assessments may offer more granularity within different functional domains and capture unique information relevant to disability progression.

Smartphones are a potential solution for remote deployment of standardized assessment protocols, which can be achieved by leveraging the smartphone-sensing technologies and apps designed to provide the patient with codified test instructions. Smartphone-based assessment offers a cost-effective and readily deployable method for monitoring neurological function remotely without the need for trained test raters or specialized monitoring devices [17,18]. This approach has already shown success in pilot and validation studies in neurological diseases, including Parkinson disease and MS [11,19-24]. However, hundreds of different SDMs can be computed from a single digital assessment, resulting in many candidate SDMs and challenges in selecting the relevant and meaningful ones, stressing the need for an SDM selection framework.

When defining a framework for the selection of smartphone-based SDMs for use in a clinical trial context, data quality and transparency are of paramount importance. First, it is essential to ensure that the sensor-derived data acquired are of high quality and that the assessment protocols are followed when deployed in an unsupervised daily living context. Second, from the initial assessment stage, it is vital to identify SDMs with the most robust metrological and statistical properties needed to obtain specific, sensitive, and responsive SDMs for future clinical trials. Although studies have demonstrated that it is feasible to use smartphone-based assessment of neurological function [8,9,11], these fundamental deliberative aspects of assessment have been only partially addressed, and the SDM selection process was not adequately described.

### Objectives

This analysis aimed to develop and apply a transparent and systematic SDM selection framework to identify robust and meaningful SDMs that can be collected remotely with smartphones and quantify cognitive and motor function in people with MS. For this purpose, we relied on data from the DigiToms study [25] and Konectom (Ad Scientiam), which is a smartphone app for collecting data on motor and cognitive function. This work intended to provide greater transparency for the selection and validation of SDMs from standardized, unsupervised, remote assessments and thereby enhance confidence in the robustness and adoption of such measurements.

## Methods

### Study Design and Participants

The DigiToms study (NCT04756700) [25] is a prospective study of people with MS and matched healthy participants. The study was conducted at CHU Hôpitaux de Bordeaux in France and classified in France as an interventional clinical study with only minimal risks and constraints. The main objectives of DigiToms were to validate DigiCog, which is a tablet-based digital version of the Brief International Cognitive Assessment for Multiple Sclerosis (BICAMS) [26,27], as a digital assessment tool for cognitive and motor functions in people with MS and to characterize the validity and reliability of SDMs obtained from Konectom in people with MS. Only the latter results are presented in this paper.

The DigiToms study recruited people with MS and healthy participants matched for age, gender, and level of education. People with MS were aged between 18 and 64 years with the diagnosis of MS according to the 2017 revised McDonald criteria [28] and an EDSS score of ≤6.0. Inclusion and exclusion criteria for both cohorts are described in Table S1 in Multimedia Appendix 1. The study assessment schedule is presented in Table 1. At clinic visit 1 (CV1), participants completed the EDSS (only patients), the daily walk test [29], either BICAMS [26] or 3 DigiCog tests [25,30], and patient-reported outcomes (PROs; Table 1). Symbol Digit Modalities Test (SDMT), a component of the BICAMS, was performed either at CV1 or clinic visit 2 (CV2), depending on randomization (Table 1). All participants first underwent a training session and then completed the full test battery under the supervision of clinic staff at CV1. Participants were initially asked to perform the digital tests daily for 14 days, but this requirement was reduced to 7 days after a scheduled interim analysis of the first 40 people with MS and 20 healthy participants showed a marked reduction in adherence after 7 days (data not shown). After the mandatory period, use was optional up to 28 days. CV2 was scheduled to occur approximately 28 days after CV1. At CV2, the conventional clinical assessments were repeated, along with a final supervised test session. The EDSS total score, and subscores were assessed by neurologists certified by the Neurostatus e-test [31].

**Table 1:** DigiToms study assessment schedule for people with multiple sclerosis.

	Day minus 2 (CV^a^0)	Day 0 (CV1)	Day 1-7 (remote visit)	Day 8-CV1 (remote visit)	CV1 minus 1-2 days (remote visit)	Day 28 (+5 or –5 days; CV2)
Information only	✓					
Inclusion and randomization		✓				
Medical history		✓				
Demographic data		✓				
MS^b^ treatments		✓				✓
EDSS^c^		✓				
PROs^d^ (ABILHAND-56, MSWS-12^e^, FSMC^f^, MSIS-29^g^)		✓				✓
DigiCog or BICAMS^h^		✓				✓
T25FW^i^ test		✓				✓
9HPT^j^		✓				✓
Konectom full battery training (no data collected)		✓				
Konectom full battery		✓	✓	✓ (optional)	✓	✓

^a^CV: clinic visit.

^b^MS: multiple sclerosis.

^c^EDSS: Expanded Disability Status Scale.

^d^PRO: patient-reported outcome.

^e^MSWS-12” 12-item Multiple Sclerosis Walking Scale.

^f^FSMC: Fatigue Scale for Motor and Cognitive Functions.

^g^MSIS-29: 29-item Multiple Sclerosis Impact Scale.

^h^BICAMS: Brief International Cognitive Assessment for Multiple Sclerosis.

^i^T25W: timed 25-foot walk.

^j^9HPT: 9-hole peg test.

### Ethical Considerations

This study was reviewed and approved by the regional French ethics committee, Committee for the Protection of Persons of the South-West and Overseas 4 (IRB Number: IORG0009855; ethics approval: CPP2020-08-071/2020-A01801-38/20.06.29.74021). Before completing any screening assessments, all study participants provided written informed consent for study participation and to authorize the use of confidential health information (including Konectom data) in accordance with national and local privacy regulations. Encryption was used for data transfer from the smartphone to a dedicated database that complied with the European General Data Protection Regulation. All study data were deidentified and aggregated for analyses presented in this paper. People with MS were not paid to participate in the study. When applicable, transport-related costs incurred by people with MS could be submitted to the investigator for review and submission to the sponsor for reimbursement. Healthy participants were compensated €75 (US $82) upon completion of the study. Compensation and reimbursement were described in the informed consent document approved by the regional French Ethics Committee.

### Digital Outcome Assessment

The Konectom digital outcome assessments tool [32] (Figure S1 in Multimedia Appendix 1), developed in collaboration between Biogen and Ad Scientiam, is an app that assesses cognitive and motor functions using a battery of tests reproducing well-established clinical assessment tools. The assessment implied three main sessions: (1) in the clinic, (2) daily activities, and (3) daily walk. The in-clinic session is supervised and follows a defined order, while the daily activities and daily walk sessions are unsupervised. The schedule of these tests is summarized in Figure S2 in Multimedia Appendix 1.

Cognitive processing speed (CPS) was measured using the CPS test, based on the validated SDMT [23]. The test consists of displaying a series of symbols on the screen (Figure S1A in Multimedia Appendix 1) and instructing participants to match symbols with their corresponding digits as quickly and accurately as possible for 90 seconds, according to a reference key displayed at the top of the screen. At each test performance, participants performed the test using either a fixed reference key, where the symbol digit pairings remained constant throughout the course of the 90-second test, or a dynamic reference key, where the symbol digit pairings changed upon the presentation of every symbol throughout the 90-second test. Only results from fixed key tests are presented in this paper.

Manual dexterity was assessed for both the dominant and nondominant hand using 2 tests previously proved to be valid and reliable in MS: the drawing test [33] and the pinching test [34]. The drawing test involves drawing predetermined shapes as quickly and accurately as possible (Figure S1B in Multimedia Appendix 1). A total of 8 shapes per hand were performed (2 attempts per shape and 4 different shapes: rectangle clockwise, rectangle counterclockwise, figure-of-8, and spiral). The pinching test involves pinching as many balloon shapes as possible within 30 seconds (Figure S1C in Multimedia Appendix 1).

Gait and postural ability were assessed using the static balance test (SBT), the U-turn test (UTT), and the daily walk test, consistently with similar efforts [22,35]. Participants were instructed to place their smartphones in a running belt at the lower back level. The SBT and UTT consisted of a 2-step test. First, participants were instructed to stand still with arms crossed and feet at hip level for 30 seconds. An auditory signal then informed the participants that they could perform 5 steps and a U-turn in a sequence repeated 5 times. Finally, in the daily walk test, participants were asked to walk outdoors and as fast as they could for 6 minutes, following a path that would allow them to walk straight for at least 250 meters.

### Data Recording

During each test, various sensors embedded in the provided and preconfigured study smartphones (iPhone X, Apple) were used to capture the necessary raw data. These sensors included screen input and coordinates (sampling frequency of 60 Hz), accelerometer, and gyroscope data (sampling frequency of 50 Hz). Assessments’ metadata were also collected, including information such as the participant’s study ID, the time stamp of each assessment, and the related session. To ensure data security and integrity, a secure protocol was used to transmit the recorded data from the smartphone to the Hébergeurs de Données de Santé servers hosted in France and managed by Ad Scientiam (Microsoft Azure).

### Data Quality Checks and Automated Detection of Deviations From Instructions

To ensure the reliability and validity of the collected data, a 3-layer quality control procedure was defined to identify and mitigate potential sources of bias or error that could influence participants’ digital test results. This process is aimed at identifying and excluding artifacts or outliers from the subsequent analysis. As part of the quality control checks, a flag was automatically raised if any anomalies or inconsistencies were detected in the different control layers.

The first layer was implemented in the app to identify assessments that did not follow the nominal scenario, such as receiving a phone call, which would automatically stop the assessment and raise a specific exit reason in the related metadata. The second layer involved identifying technical issues with the data capture process at the sensor level, such as confirming the presence and the consistency of the expected data with hardware specifications (eg, expected and stable sampling frequency). The third layer leveraged previously developed and publicly available [36] algorithms to automatically identify critical deviations from the assessment instructions, potentially occurring in unsupervised remote assessments. In particular, the following deviations from the instructions were identified as critical and used to identify signals or measures to be excluded from the analysis: smartphone not worn in the belt during the SBT, UTT, and daily walk test; presence of excessive extra motions during the SBT; absence of U-turns during the UTT; finger lifting during the drawing test; smartphone not kept in portrait mode during the drawing and pinching tests; incomplete drawing or drawing path length outside expected values during the drawing test; and missing attempts at the pinching tests.

### SDM Computation and Selection

SDMs were computed for each test using a purposely developed and publicly available Python library [36]. To select promising SDMs for our context of use, a previously proposed selection process [37] was adopted after tailoring it to the study needs. In more detail, relevant literature was reviewed, and a first set of measures per digital test was identified, which was expected to capture meaningful concepts of interest in people with MS and have high interpretability. For the pinching and drawing tests, only SDMs for the dominant hand were included to focus on the most relevant information. Subsequently, the statistical properties of the SDMs were evaluated to establish their suitability to be collected in unsupervised settings and potentially serve as accurate and sensitive outcomes in people with MS. Specifically, 2 experts (CM and CK) first independently and blindly evaluated the distribution and potential systematic bias of each original SDM and of the versions of the SDMs obtained after applying variance-stabilizing transformations (logarithmic, square root, and reciprocal functions). For this purpose, quantile-quantile and Bland-Altman plots were visually inspected to identify the original or transformed SDMs that most closely followed a normal distribution and did not show systematic bias. After jointly reviewing the contrasting results, the assessors discarded SDMs that showed systematic bias or significantly deviated from a normal distribution, such as those affected by ceiling or floor effects, low resolution, or outliers. These steps ensured the overall robustness of the selected SDMs and allowed to fulfill the basic assumption for subsequently calculating statistical performance metrics, namely, the intraclass correlation coefficient (ICC 3, k) [38] and the minimal detectable change at 95% CI (MDC95%) [39,40]. The ICC accounts for the interparticipant and intraparticipant variability of a measure, with an ideal measure being stable within a participant but discriminating behavior between participants [41]. The MDC95% provides a range of values in which it is not possible to discriminate between statistical noise and physiological change. This implies that, in a longitudinal study, changes in measures would only be deemed statistically meaningful if they exceed the MDC95% threshold [42]. To enable comparability across SDMs, the MDC95% is normalized to the range of a measure and ultimately expressed as a proportion or percentage value. Instead of applying strict predefined thresholds for the SDM selection framework, ICC, MDC95%, and the other available evidence (literature, hypotheses, interpretability, flags raised from data quality checks, and prior experience with SDMs) were used in this study to select a final set of SDMs per test. This approach allowed for more flexibility in the SDM selection process while still enabling a hypothesis-driven and data-driven selection of SDMs.

This framework and all subsequent statistical analyses were performed using only data from people with MS who had performed at least 7 remote instances of a given test with at least 2 valid remote instances of a given SDM*.*

### Statistical Analysis

Additional analyses were performed to describe the properties of the SDMs that were selected through the SDM selection framework.

#### Reproducibility of SDMs

The reproducibility of the SDMs was evaluated with ICC and MDC95% (remote SDM only) as well as by calculating the mean relative difference (MRD) between SDMs from supervised versus unsupervised Konectom assessments [42]. ICC was calculated based on a mixed-effect model with subject-level random effects, and an additional fixed effect of visit number was added to account for potential practice effects. The MDC95% was calculated as 1.96 × square root of 2 within-subject variance, with the within-subject variance derived from the intermediate results of the corresponding ICC values. The relative (percentage) MDC95% was calculated as the MDC95% of an SDM divided by the range of its distribution. The MRD between SDMs from supervised versus unsupervised Konectom assessments was calculated as the average of the differences between the first remote instance of an SDM and the in-clinic SDM at CV1, divided by the SDM values at CV1.

Test-retest reproducibility was considered poor (ICC<0.5), moderate (ICC=0.5-0.75), good (ICC=0.75-0.9), or excellent (ICC>0.9) [41].

#### Convergence of SDMs With Clinical Anchors, EDSS, and PROs

The convergence of selected SDMs with domain-specific clinical anchors (oral SDMT, 9-hole peg test, 9HPT; and timed 25-foot walk, T25FW test); EDSS; and domain-relevant subscores, with domain-relevant PROs, was evaluated with Spearman correlation coefficients (ρ). The convergence was considered as good-to-excellent if |ρ|>0.75, moderate-to-good if 0.5<|ρ|≤0.75, fair if 0.25≤|ρ|<0.5, and not correlated if |ρ|<0.25 [43]. The 9HPT tested the average time to complete the test from both attempts (seconds), the T25FW test tested the average time to complete the test from both attempts (seconds), and SDMT tested the number of correct responses.

## Results

### Study Participants’ Baseline Characteristics

Between October 2020 and July 2022, a total of 88 people with MS and 70 healthy participants were enrolled in the study. Three people with MS and 2 healthy participants were either lost to follow-up or withdrew their consent, and their data were excluded from further analyses. Study participants’ baseline characteristics are presented in Table S2 in Multimedia Appendix 1. As intended, people with MS and healthy participants were well balanced overall for the matching criteria (age, gender, and education) as well as for BMI and hand dominance. The level of disability of the people with MS was mild (median EDSS score of 2, IQR 1.5-3.0), and none of the patients required an assisting device to walk. Compared with healthy participants, people with MS performed worse on 9HPT, T25FW test, daily walk test, and SDMT. The EDSS score distribution and treatment allocation are presented in Figure S3 and Table S3 in Multimedia Appendix 1, respectively. No safety issues or disease relapses were reported during the observation period.

### Adherence to Protocol and Test Instructions

**Figure 1:**
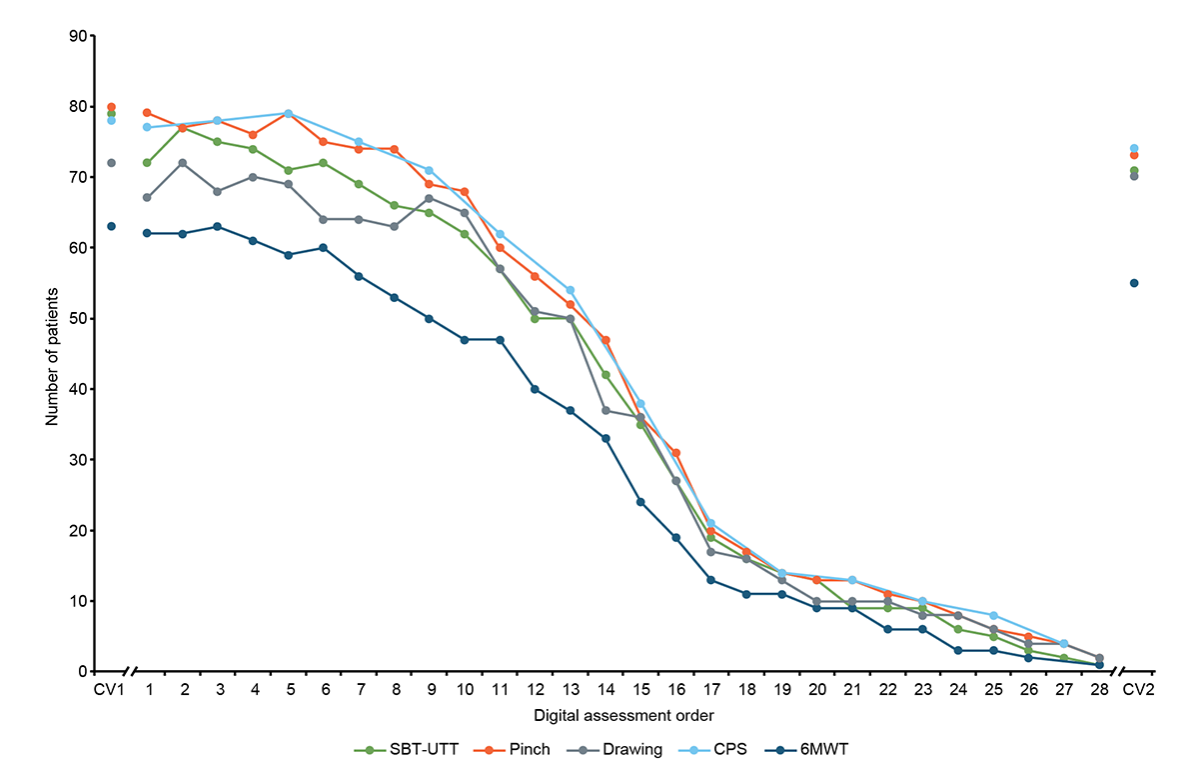
Number of people with multiple sclerosis performing a complete Konectom test during clinic visit 1 (CV1), first to 28th remote sessions, and clinic visit 2 (CV2). 6MWT: 6-minute walk test; CPS: cognitive processing speed; SBT: static balance test; UTT: U-turn test.

Among the 85 people with MS, the number of those completing at least 7 full remote test batteries ranged from 56 to 75 (66%-88%), which then decreased in the following period (Figure 1). People with MS took a median of 8 (IQR 7-26) days to complete the first 7 remote instances of the digital tests.

Instances were flagged when the participants deviated from the test instructions provided by the Konectom app. The percentage of instances with deviations from Konectom test instructions was low (an average of 6.2% across tests, SD 4.4%), and no systematic deviation patterns in performing the tests were identified for the people with MS (Table 2).

**Table 2:** Summary of the number and frequency of instances with deviations from the Konectom test instructions that were automatically detected through dedicated algorithms in the DigiToms study participants (N=153).

Test	Total instances, n	Instances with deviations from test instructions, n (%)	Total users, n (%)	Users with at least 1 instance with deviations, n (%)
Daily walk	2252	103 (4.6)	151 (98.7)	27 (17.9)
CPS^a^	2603	0 (0)	153 (100)	0 (0)
Pinching	5140	14 (0.3)	153 (100)	9 (5.9)
SBT^b^	2546	390 (15.3)	153 (100)	105 (68.6)
UTT^c^	2546	193 (7.6)	153 (100)	40 (26.1)
Drawing an infinity shape^d^	9428	812 (8.6)	153 (100)	107 (69.9)
Drawing a rectangle clockwise^e^	9428	553 (5.9)	153 (100)	87 (56.9)
Drawing a rectangle counterclockwise^d^	9428	432 (4.6)	153 (100)	87 (56.9)
Drawing a spiral^d^	9428	812 (8.6)	153 (100)	120 (78.4)

^a^CPS: cognitive processing speed.

^b^SBT: static balance test.

^c^UTT: U-turn test.

^d^For both attempts of both hands.

### SDM Selection

**Figure 2:**
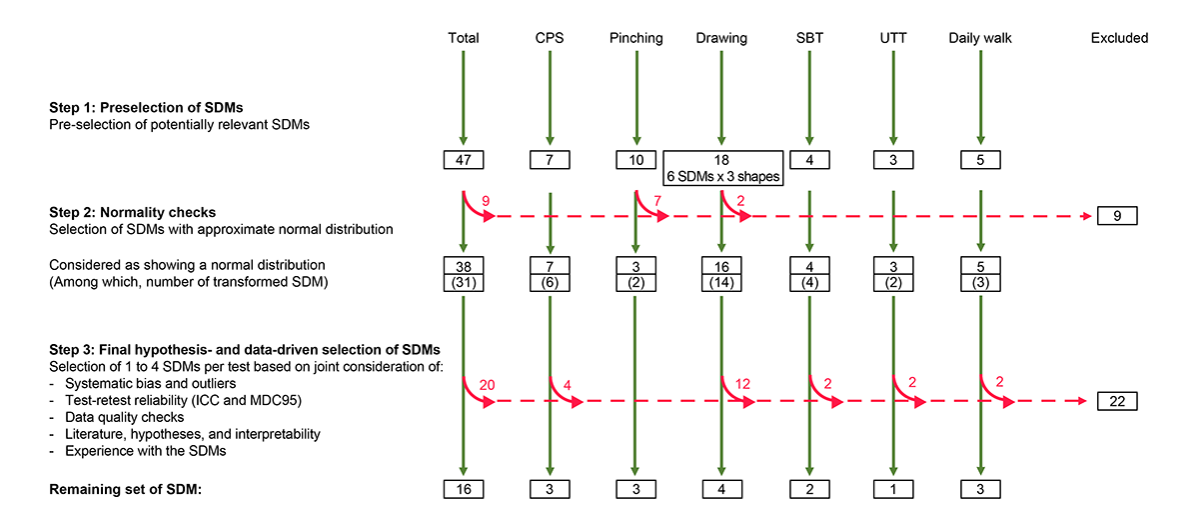
Sensor-derived measurement selection overview. CPS: cognitive processing speed; ICC: intraclass correlation coefficient; MDC95%: minimal detectable changes at 95% CI; SBT: static balance test; SDM: sensor-derived measure; UTT: U-turn test.

The conceptual framework for optimal SDM selection and analysis is presented in Figure S4 in Multimedia Appendix 1. In total, 16 (34%) of the initial 47 SDMs passed the SDM selection process. Results of the intermediate steps of the selection are presented in Figure 2, while detailed definitions of the selected SDMs and their descriptive statistics are summarized in Table 3. Further details on the discarded SDMs are presented in Multimedia Appendix 2.

**Table 3:** Selected sensor-derived measure (SDM) characteristics from Konectom assessments performed in people with multiple sclerosis and healthy participants, with corresponding measure description, type of transformation performed, and descriptive statistics.

Test and SDM (unit^a^)		Description	Transformation	People with multiple sclerosis			Healthy participants			*P* value^b^
				Value, n	Value, median (IQR)	Value, n		Value, median (IQR)		
**CPS^c^**										
	Correct responses (unitless)	Total number of correct responses for the fixed key sets during the symbol-to-digit phase	None	74	44.5 (24-67)	66		51 (33-64)	<.001	
	Reaction time median (s)	Median reaction time of the correct responses’ distribution during the symbol-to-digit phase	Reciprocal function	74	0.59 (0.32-0.97)	66		0.68 (0.47-0.93)	<.001	
	Reaction time unique keys (s)	Median reaction time of the correct responses for unique keys only. A key is unique if it cannot be associated with any other key in the header	Reciprocal function	74	0.74 (0.31-1.17)	66		0.82 (0.55-1.25)	.004	
**Pinching**										
	Total number of pinched balloons (unitless)	The number of successful pinches, where a successful pinch is a screen interaction with at least 2 fingers at the screen that leads to the target balloon bursting	None	76	5 (0-8)	66		6 (0-8)	.003	
	Contact distance (point)	Median distance between the contact point of bottom finger and balloon appeared at the start of a pinch attempt	Sqrt^d^ (x)	76	6.14 (2.73-10.12)	66		5.96 (3.36-10.34)	.59	
	Pinching speed (point/s)	Median speed of the top finger during pinch attempts	Sqrt (x)	76	0.59 (0.16-1.06)	66		0.59 (0.29-0.92)	.89	
**Drawing**										
	Duration-normalized accuracy (spiral; 1/[point × s])	Drawing accuracy is normalized by the time spent between the first and last interaction of the participant with the screen. Accuracy is 1 over the similarity between reference shape and drawn shape measured using dynamic time warping; it is calculated on the spiral shape	None	67	0.03 (0.01-0.08)	58		0.04 (0.02-0.08)	.16	
	Speed (spiral; point/s)	Median of instantaneous drawing speed. The feature is calculated on the spiral shape	Sqrt (x)	67	19.13 (12.58-28.83)	58		18.99 (11.14-33.28)	.51	
	Duration (spiral; ms)	Time spent between the first and last interaction of the participant with the screen; it is calculated on the spiral shape	Log x	67	8.55 (7.77-9.43)	58		8.61 (7.50-9.70)	.44	
	Speed variability and square (unitless)	Coefficient of variation of instantaneous drawing speed; it is calculated on the square shape	None	69	0.54 (0.36-0.81)	59		0.50 (0.34-0.67)	.02	
**SBT^e^**										
	Area of 95% confidence ellipse (microG^2^)	Area of the ellipse containing 95% of accelerations belonging to the mediolateral or anteroposterior plane from 5 to 30 s	Log x	55	3.02 (1.07-6.33)	57		2.38 (1.17-3.99)	<.001	
	Jerk (mG/s)	Jerk of measured accelerations in the anteroposterior or medio-lateral plane from 5 to 30 s	Reciprocal function	55	0.07 (0.02-0.15)	57		0.10 (0.05-0.19)	.002	
**UTT^f^**										
	Turning speed (rad/s)	Mean absolute angular velocity recorded during the turn (median over first 4 turns)	None	75	3.77 (2.26-6.19)	66		4.01 (2.24-5.58)	.26	
**Daily walk test**										
	Step power (m^2^/s^3^)	Integral of the mean-centered acceleration magnitude signal over a step	Log x	61	1.67 (0.48-2.79)	62		1.83 (0.52-2.83)	.25	
	Step regularity (unitless)	First peak of autocorrelation function of the resultant acceleration	None	61	0.78 (0.46-0.93)	62		0.79 (0.46-0.89)	.48	
	Stride regularity (unitless)	Second peak of autocorrelation function of the resultant acceleration	None	61	0.86 (0.62-0.93)	62		0.85 (0.59-0.91)	.44	

**^a^**Before transformation.

^b^*P* values for sensor-derived measure differences between people with multiple sclerosis and healthy participants were obtained from Wilcoxon test.

^c^CPS: cognitive processing speed.

^d^Sqrt: square root.

^e^SBT: static balance test.

^f^UTT: U-turn test.

### Reproducibility

#### MRD Between First Remote Versus CV1 Konectom SDMs

**Figure 3:**
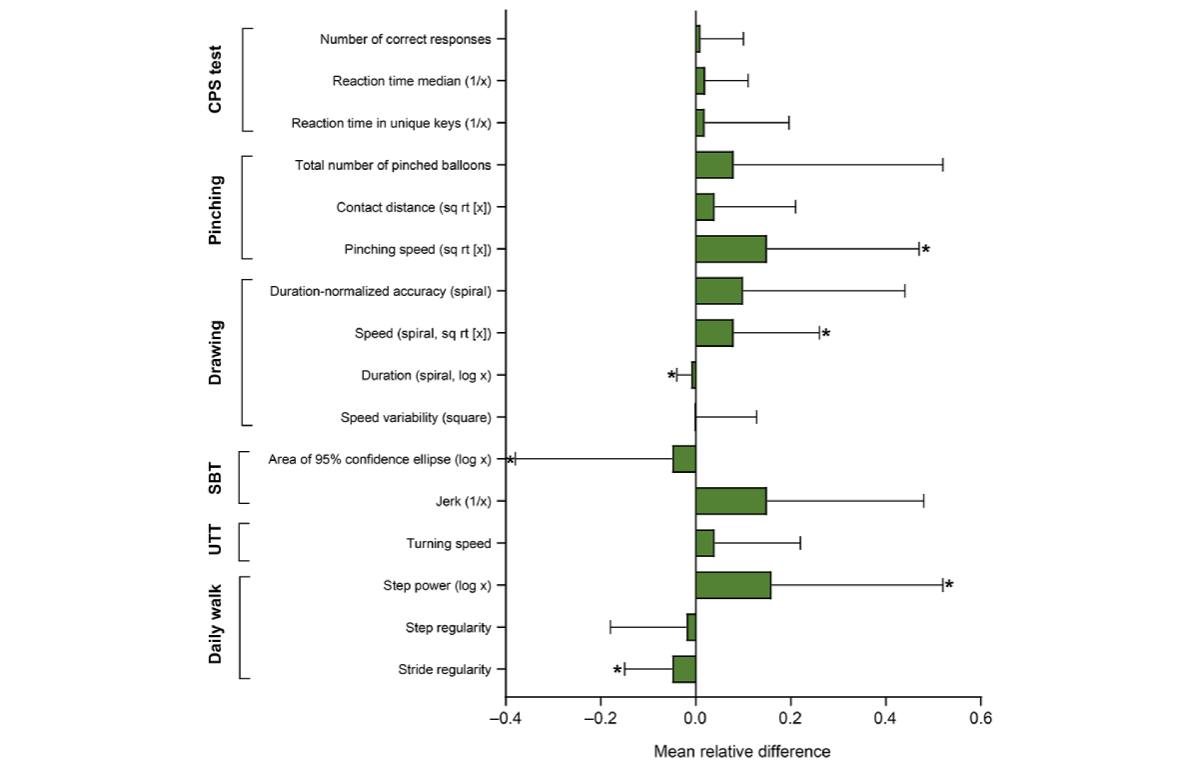
Mean relative differences between sensor-derived measures (SDMs) from supervised in-clinic versus unsupervised remote Konectom assessments performed in people with multiple sclerosis, calculated as the average of the differences between the first remote instance of an SDM and the in-clinic SDM at clinic visit 1 divided by SDM values at clinic visit 1. The error bars represent standard deviation. *P<.05. CPS: cognitive processing speed; SBT: standing balance test; UTT: U-turn test.

Changes associated with performing the test without supervision in a free-living environment are shown in Figure 3, reporting the MRD between CV1 and the remote assessments. Most differences were not significant (*P*≥.05) and were <10%.

#### Reproducibility of the First 7 Remote Konectom SDMs

**Figure 4:**
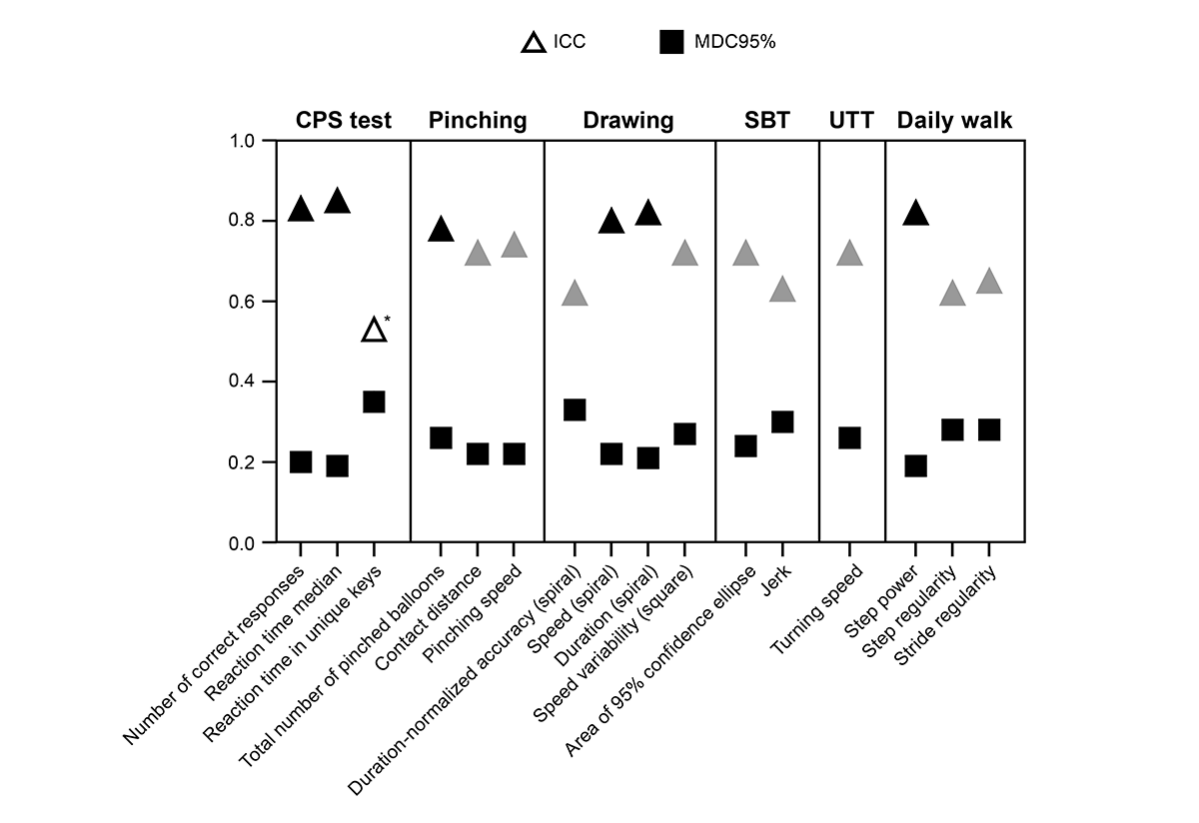
Sensor-derived measure (SDM) reproducibility for the first 7 remote instances of Konectom sessions in people with multiple sclerosis assessed by intraclass correlation (ICC) and minimal detectable change at 95% CI (MDC95%). ICC ranged from moderate to good; moderate (ICC = 0.5-0.75), or good (ICC = 0.75-0.9). MDC95% for each SDM is represented relative to the range of its distribution (eg, 0.2 signifies that the minimal detectable change threshold is at 20% of the full range of SDM distribution). *P>.05. CPS: cognitive processing speed; SBT: static balance test; UTT: U-turn test.

Overall, selected measures had ICC values ranging from 0.53 to 0.85 (moderate-to-good) [32] and MDC95% ranging from 19% to 35%, based on the first 7 test performances. For balance and ambulation measures, ICC values ranged from 0.62 to 0.82, with MDC95% between 19% and 30%. For upper extremity SDMs, ICC values ranged from 0.62 to 0.82, with MDC95% between 21% and 33%. For cognition measures, ICC ranged from 0.53 to 0.85, with MDC95% between 19% and 35% (Figure 4).

### SDMs’ Convergence With Clinical Measures and PROs

#### Correlations With Relevant In-Clinic Benchmarks of MS Functional Performance Outcomes: SDMT, 9HPT, and T25FW Test

**Figure 5:**
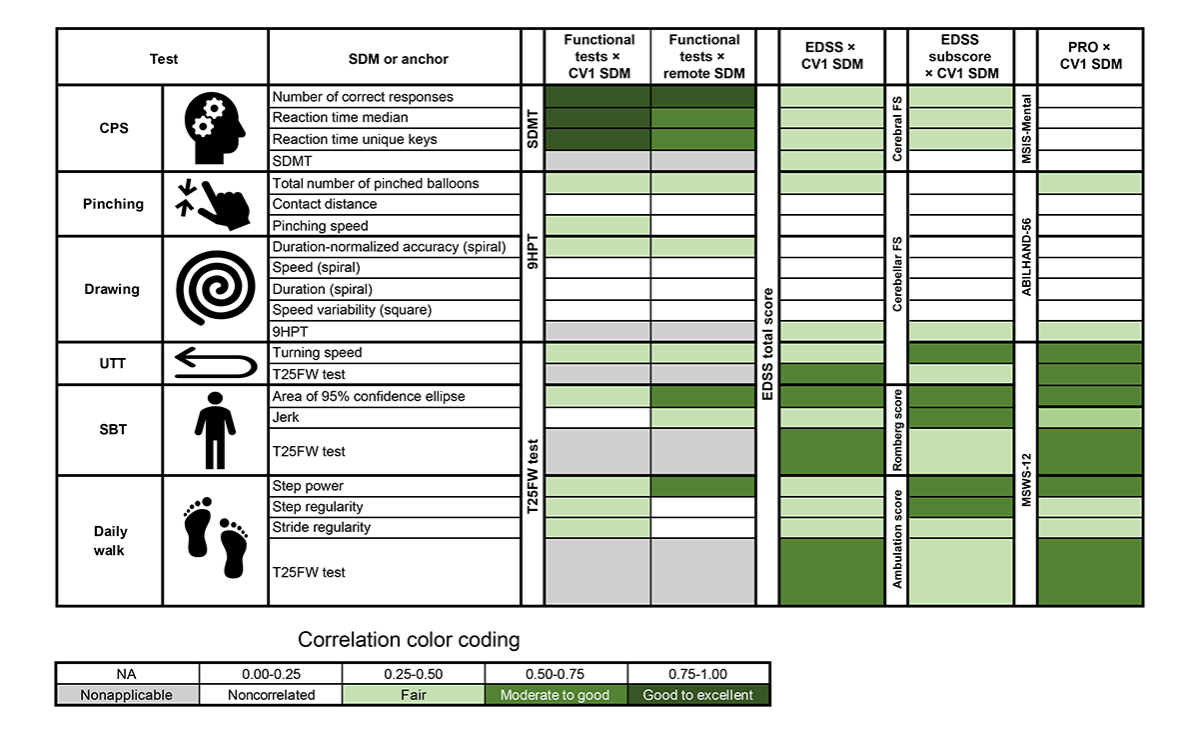
Correlations between conventional in-clinic measures and Konectom sensor-derived measures (SDMs). SDM convergence with conventional clinical measures and patient-reported outcomes (PROs) in people with multiple sclerosis are displayed. EDSS: Expanded Disability Status Scale; FS: functional score; MSIS-29: 29-item Multiple Sclerosis Impact Scale; MSWS-12: 12-item Multiple Sclerosis Walking Scale.

Figure 5 shows the correlations that were assessed between (1) the SDMT and CPS test SDMs; (2) the 9HPT and SDMs from the drawing and pinching tests; and (3) the T25FW test and SDMs from the SBT, UTT, and daily walk test. Correlations were in the expected direction; that is, increasing levels of MS-related disability as measured by SDMT, 9HPT, or T25FW were associated with poorer performances on each corresponding Konectom SDM. As expected, a good-to-excellent convergence was observed between the SDMT and CPS test SDMs. All selected SDMs extracted from the pinching and drawing tests had a rather fair convergence with the 9HPT, except the contact distance in the pinching test and the speed variability in the unsupervised drawing shape (square), which did not correlate with the 9HPT. All selected SDMs extracted from the SBT, UTT, and daily walk test also had a rather fair convergence with the T25FW test, except the daily walk test stride regularity in the clinic, which did not correlate with the T25FW test. Overall, correlations were similar when tests were performed in a clinic or remotely.

#### Correlations With Corresponding PRO and EDSS Functional Score and Subscores

When significant, correlations between selected SDMs from Konectom assessments (in the clinic at CV1) and the EDSS, relevant EDSS functional scores or subscores, and relevant PROs (Figure 5) were all in the expected direction. In the domain of cognition, all 3 CPS test SDMs had a fairly significant convergence with overall EDSS and its cerebral functional system (FS) score, which numerically outperformed that of the SDMT. Neither CPS test SDMs nor SDMT correlated with the 29-item Multiple Sclerosis Impact Scale mental subscore. In the domain of manual dexterity, among all selected SDMs of the pinching and drawing tests, only the total number of pinched balloons in the pinching test had a fair convergence with EDSS and ABILHAND-56 but not with the cerebellar FS score. In the domain of gait and balance, all selected SDMs from the SBT, UTT, and daily walk test had a fair to moderate-to-good convergence with the 12-item Multiple Sclerosis Walking Scale, the overall EDSS, and the appropriate EDSS subscore (the Romberg test, cerebellar FS score, or ambulation scores).

## Discussion

### Principal Findings

Digital health technologies (DHTs) and SDMs of motor and cognitive function are promising solutions for complementing currently limited clinical assessments of disease status and progression in people with MS. However, their development, validation, and acceptance require complex steps, including selecting the most reliable and relevant SDMs. The purpose of this work was to develop and apply a transparent and systematic SDM selection framework to identify robust, hypothesis-driven, and relevant SDMs that can be collected remotely with smartphones and objectively assess cognitive and motor functions in people with MS for continued clinical validation. The framework was based on the hypothesis-driven motivation of SDMs with high interpretability; the evaluation of remote data quality (including automated detection of deviations from test instructions); and the evaluation of relevant statistical properties, including normality (quantile-quantile plots), test-retest reliability (ICC), bias (Bland-Altman plots), and MDC95%. Importantly, in this study, we report all 47 initially considered SDMs and their properties, along with the 16 (34%) most promising SDMs selected for assessing motor and cognitive function, which were identified using the systematic SDM selection framework. We advocate other researchers to follow similar transparent and systematic reporting to ensure that the field can reach standardization and a consensus on smartphone-based SDMs to be used in the assessment of people with MS, which can help facilitate the adoption of DHTs in clinical trials and support conversations with regulators. It is also worth highlighting potential cost advantages to adopting valid and reliable SDMs to support clinical trial design. Sample size and trial duration are important cost drivers for clinical trials. High-frequency sensor-based remote data collection can reduce sample size requirements by increasing precision and reducing measurement variability [44,45]. A robust framework such as the one proposed in this study can enable the selection of high-quality SDMs with favorable statistical properties for clinical validation and subsequent use to enhance trial design and potentially reduce cost. While other SDM selection frameworks have been used in the literature [37,46,47], these are not tailored to address the challenges with data that have been collected remotely in unsupervised settings with smartphones. Therefore, this work makes an important contribution to mature the field of smartphone-based digital biomarker development and helps to identify promising SDMs of motor and cognitive function that can further be validated in longitudinal MS trials.

Adherence to protocol and test instructions is necessary to gather sufficient high-quality SDM data in a remote, unsupervised setting, whereas nonadherence to study protocol (ie, test not performed) affects study power through missing values, deviations from test instructions (ie, test not performed as intended), and increased noise in digital measures. However, descriptions of adherence to both the study protocol and test instructions are often lacking in the literature on smartphone-based assessments. Although adherence was observed to decline after 7 days, evaluation of adherence to protocol and test instructions showed that most participants performed 7 complete assessments within the 8 days following the first CV1 and that the occurrence of deviations from test instructions was low across all tests. This confirms previous reports that smartphones are indeed suitable DHT tools to collect high-quality remote SDM data in people with MS [48], even though these results should be confirmed across a broader MS population with a wider range of disabilities.

In this study, the selection framework allowed for an unbiased evaluation of different potential SDMs of balance, ambulation, manual dexterity, and cognition. On the basis of our framework and subsequent analyses, step power was the most promising SDM for balance and ambulation, showing higher ICC and lower MDC95% values than more traditional SDMs, such as step duration or step variability, which also showed higher mean absolute errors and biases across remote visits. This latter result might be associated with variations in the walking context chosen by the patients (eg, including stops at traffic lights and crowded paths), but additional contextual data would be needed to test this hypothesis. For the pinching test, 3 SDMs of pinching duration, pinching speed, and contact distance were selected, as accuracy and smoothness measures were less robust and the double touch asynchrony measure had strong ceiling or floor effects, potentially due to the limited sampling rate of smartphones. For the drawing test, 4 measures of speed, speed variability, duration, and duration-normalized accuracy were chosen, as they outperformed measures of movement smoothness (jerk) and accuracy. Three of these measures were extracted from the spiral drawing test, as it is the clinically most-accepted shape and showed sufficient clinimetric properties. Jerk-based smoothness measures have traditionally been analyzed in the context of goal-directed ballistic arm movement tasks, and which smoothness measures are suitable for the drawing and pinching task remains to be explored [49]. For the CPS test, most SDMs were highly correlated with one another and showed comparable statistical properties. The median reaction time had similar statistical metrics compared with the total number of correct responses, the classical output of an SDMT. Despite lower ICC and higher MDC95% values, the unique key response time was selected for its potential ability to explore a different concept of interest because unique keys do not have symmetrical counterparts that may confuse the participant.

SDMs obtained in a supervised, in-clinic context compared with unsupervised, remote SDMs collected in a free-living environment may measure different constructs (performance vs ability, respectively). Differences between SDMs collected in a supervised and unsupervised environment have been demonstrated in MS and other populations, notably with different impacts of supervision across different gait measures and populations [50]. In this study, statistically significant differences between first in-clinic and remote tests were observed in some SDMs; overall, correlation patterns between SDMs and relevant in-clinic benchmarks of MS functional performance outcomes were similar when data were collected in the clinic or remotely. Differences were most apparent for ambulation-related SDMs from the daily walk test. This is not surprising given the change in walking course that would be expected to occur when moving the test session from a well-controlled in-clinic visit to unsupervised remote settings. Despite this finding, there was generally moderate-to-good test-retest reliability in the remote setting across all selected SDMs, which reassures that reliable SDM data can be obtained remotely using the Konectom assessment tool. Longitudinally, the differences between measures collected in a supervised and unsupervised environment are important to understand in the context of a given study and measurement paradigm, especially if both will be used.

The test-retest reliability (ICC) of the selected SDMs across the first 7 remote assessments was moderate to good, while the MDC95% ranged from 19% to 35%. Although the measurement paradigm was different, for comparison, a recent, large-scale analysis of traditional clinical end points from clinical trials showed similar ICCs: 0.71 for the T25FW test and 0.84 for 9HPT from 2 to 6 test repetitions in people with MS [51]. In terms of what can be considered a meaningful change, a 20% change in the T25FW test and 9HPT is commonly used as a heuristic threshold, while MDC95% of 21% to 36% and 12% to 30% have been reported, respectively [52]. For remotely acquired SDMs of cognition, such as the number of correct responses and the median reaction time of the correct responses in the CPS test, ICCs were 0.83 and 0.85, with MDC95% of 20% and 19%, respectively. The study by Goldman et al [51] showed an ICC of 0.85 for SDMT, with another study showing an ICC of >0.9 across the span of 1 week [40]. Appropriate thresholds for change are less well established for cognition and SDMT. Frequently, a 4-point change (10%) has been cited as a threshold for SDMT [23,53,54]; however, the stability of cognitive change based on this threshold and even a 20% threshold has been questioned. More work is needed in this respect [8,55]. Although, by design, we are using a different measurement paradigm, our ICCs and MDC95% for selected SDMs extracted from remote unsupervised assessments are aligned with what is reported for conventional in-clinic measures in the literature. An important caveat is that this is likely to vary with the disability of the population and should be examined in future studies in populations with a wider range of disabilities. Altogether, over the first 7 remote test performances, the selected SDMs demonstrated good measurement properties, likely as a result of the framework used for their selection. These measurement properties may improve with a greater number of data points collected in a longitudinal setting that would enable the implementation of different aggregation methods to enhance the signal-to-noise ratio.

Selected SDMs showed a range and diversity of correlation patterns with clinical anchors, suggesting that the Konectom tests and some of the SDMs capture aspects of cognition and upper and lower limb motor function similar to conventional in-clinic functional performance measures, while others may capture uniquely nonoverlapping aspects of those functions. For example, it is not surprising that SDMs from the CPS test and the SDMT showed the greatest convergence, given the similarity in the test construct and design. Conversely, other SDMs (in particular, those derived from the pinching or drawing tests and the daily walk test) showed a weaker correlation against the clinical assessments 9HPT and T25FW test, respectively. It should be noted that the drawing test may rely to a greater extent on fine motor hand and finger function and to a lesser extent on executive strategy compared with the upper limb assessment by the 9HPT. The daily walk test and the conventional T25FW test also represent different test constructs, with different walking and balance control mechanisms involved in gait initiation and short walking, which comprise the T25FW test, as opposed to those associated with a longer 6-minute walking paradigm [51]. Further study of these novel SDMs as extracted from the Konectom digital outcome assessments tool in a longitudinal setting is warranted to understand their potential value in increasing the accuracy and sensitivity of MS disability progression measures in those key domains of functional performance impairment.

Generally, selected SDMs showed similar correlation strength with total EDSS and domain-relevant EDSS subscores compared with the SDMT, 9HPT, and the T25FW test. The CPS SDMs showed a slightly greater correlation with the total EDSS than the SDMT, whereas the SDMs from the drawing and pinching tests were more weakly correlated with the EDSS than the 9HPT. Interestingly, the SDMs showed at least similar correlations with PROs than the SDMT, 9HPT, or T25FW test anchors, suggesting that they might capture concepts of interest with similar levels of relevance to patients.

The data from this study and analyses should be interpreted in the context of the following caveats. The first is the small number of patients, albeit our study sample size was on par with prior cross-sectional validation studies [8,9,11]. The second is the overall low level of disability; the DigiToms patient population had a relatively low level of physical disability and functional MS-related impairment as defined by baseline EDSS, SDMT, T25FW test, and 9HPT. While it may be informative to assess SDMs in patients with a wider range of impairments, the reported results are encouraging and may support future use of SDMs in the setting of early interventions. The third is that these analyses were cross-sectional in nature. While cross-sectional data provide useful information, further characterization of measurement properties in longitudinal trials would be needed to prove sensitivity to changes in the selected SDMs.

### Conclusions

This study marked an important step in the pathway toward acceptance of quantitative methods for objective and more frequent assessment of motor and cognitive functions in patients with MS. After having shared in a previous study the Python library adopted for the calculation of SDMs [36], in this paper, we presented a framework for their optimal selection and analysis, with the goal of further enhancing transparency and confidence in the robustness of SDMs from smartphone-based tests. While their sensitivity to changes will have to be proven in longitudinal interventional studies, the reliability and convergent validity of the selected measures presented in this study are an essential milestone for acceptance and adoption in clinical trials and, ultimately, more accurate and sensitive measures of cognitive and motor function in people MS. To support further use of DHTs in clinical trials, we advocate that researchers adopt similar standardized frameworks to aid selection of high-quality reproducible SDMs.

## Appendix

Multimedia Appendix 1 DigiToms inclusion and exclusion criteria, participant baseline characteristics and baseline disease modifying therapy treatment allocation, Expanded Disability Status Scale distribution, Konectom app test description and schedule, and process for selection of sensor-derived measures.

Multimedia Appendix 2 Sensor-derived measure full and selected feature descriptions.

## Abbreviations

9HPT: 9-hole peg test
BICAMS: Brief International Cognitive Assessment for Multiple Sclerosis
CPS: cognitive processing speed
CV1: clinic visit 1
CV2: clinic visit 2
DHT: digital health technology
EDSS: Expanded Disability Status Scale
ICC: intraclass correlation coefficient
MDC95%: minimal detectable change at 95% CI
MRD: mean relative difference
MS: multiple sclerosis
PRO: patient-reported outcome
SBT: static balance test
SDM: sensor-derived measure
SDMT: Symbol Digit Modalities Test
T25FW: timed 25-foot walk
UTT: U-turn test

## Data Availability

The datasets generated during and analyzed during this study are available from the corresponding author on reasonable request.
