# Supplementary material for: Sensor-Derived Measures of Motor and Cognitive Functions in People With Multiple Sclerosis Using Unsupervised Smartphone-Based Assessments: Proof-of-Concept Study
Source: JMIR Form Res. 2024 Nov 8;8:e60673. doi: 10.2196/60673 (PMC11584543; doi:10.2196/60673)
Supplement: Multimedia Appendix 1 [file formative_v8i1e60673_app1.pdf]

Multimedia Appendix 1

Table of Contents

Table S1. Inclusion and exclusion criteria of the DigiToms study. 2

Table S2. Study participants' baseline characteristics. 3

Table S3. DMT treatment allocation. 5

Figure S1. Konectom™ app tests for cognitive and manual dexterity function. 6

Figure S2. Summary schedule of Konectom™ app tests for cognition, dexterity, and gait and posture. 7

Figure S3. EDSS distribution. 8

Figure S4. Process for selection and analysis of sensor-derived measures. 9

Table S1. Inclusion and exclusion criteria of the DigiToms study.

	**Inclusion criteria**	**Exclusion criteria**
People with MS	• Aged 18-64 years<br>• Diagnosis of MS according to 2017 revised McDonald criteria<br>• EDSS score ≤6.0	• Severe ongoing psychiatric condition, including depression<br>• Change of DMT or psychotropic treatment in the last month<br>• Clinically significant addiction<br>• Recent acute relapse or disability worsening (<1 month)<br>• Steroid course in the previous month<br>• Neuropsychological testing in the previous year
Healthy participants	• Recruited from a center-owned database<br>• Participants were considered healthy if they were not suffering from injuries that could affect walking and turning, or fine motor function<br>• Aged 18-64<br>• Matched to MS patient sample for age, gender, and level of education	• Severe ongoing psychiatric condition, including depression<br>• Any clinically significant neurological disorder<br>• Any psychotropic therapy consumption<br>• Clinically significant addiction<br>• Previous participation in other cognitive study using the same tests<br>• Developmental learning disabilities

DMT disease-modifying therapy. *EDSS* Expanded Disability Status Scale. *MS* multiple sclerosis.

Table S2. Study participants' baseline characteristics.

Characteristic	People with MS (n=85)	Healthy Participants (n=68)	*P* value
**Age** (year), median (min, max)	44.0 (20.0, 62.0)	40.5 (20.0, 62.0)	.3018[a]
**Gender,** n (%)			
Female	70 (82.4)	57 (83.8)	.8098[b]
Male	15 (17.6)	11 (16.2)	
**Body mass index (kg/m^2),** median (min, max)	23.7 (16.6, 44.6)	23.5 (16.8, 36.5)	.9269[a]
**MS type,** N (%)			
PPMS/PRMS/SPMS	4 (4.7)	NA	NA
RRMS	81 (95.3)	NA	NA
**Disease duration** (year), median (min, max)	8 (0,37)	NA	NA
**Number of relapses last 12 months, median (min, max)**	0.0 (0.0, 5.0)	NA	NA
**Education Level,**[f] N (%)			
ISCED <3	14 (16.5)	9 (13.2)	.9374[b]
ISCED 3	16 (18.8)	12 (17.6)	
ISCED 4-6	38 (44.7)	32 (47.1)	
ISCED >6	17 (20.0)	15 (22.1)	
**Job status**, N (%)			
Unemployed	18 (21.2)	6 (8.8)	.0057[b]
Part-time job	22 (25.9)	9 (13.2)	
Full-time job	45 (52.9)	53 (77.9)	
**Dominant hand**, N (%)			
Left	9 (10.6)	5 (7.4)	.4904[b]
Right	76 (89.4)	63 (92.6)	
Ambidextrous	0 (0.0)	0 (0.0)	
**EDSS,** median (min, max)	2.0 (0.0, 4.5)	NA	NA
**9HPT** dominant hand (seconds),[d] mean (SD)	21.4 (6.7)	18.0 (2.0)	<.0001[a]

**9HPT** nondominant Hand (seconds),[d] mean (SD)	22.2 (4.4)	18.6 (2.0)	<.0001[a]
**T25FW** (seconds),[e] mean (SD)	4.5 (0.8)	4.0 (0.5)	<.0001[a]
Daily Walk test (meters), mean (SD)	495.0 (102.3)	556.1(77.7)	.0002[a]
**SDMT** (number of correct responses),[c] median (min, max)	58 (29, 85)	65 (41, 85)	.0002[a]
**BVMT**[c], median (Q1, Q3)	31 (6, 36)	32 (10, 36)	.1155[a]
**FVLT**[c], median (Q1, Q3)	68 (34, 77)	67.5 (38, 77)	.6445[a]

[a]Wilcoxon test.

[b]Chi-square test.

[c]Performed either at V1 or V2, depending on randomization.

[d]Average time to complete the test from both attempts.

[e]Average time to complete the test from both attempts (seconds).

[f]ISCED <3: No baccalaureate = did not complete upper secondary education, ISCED 3: Baccalaureate = completed upper secondary education, ISCED 4-6: Secondary education short (≥ Baccalaureate +2) = post-secondary tertiary education < master level, ISCED >6: Secondary education long (≥ Baccalaureate +5) = master level and above.

BVMT Brief Visuospatial Memory Test. *EDSS* Expanded Disability Status Scale. *FVLT* French Verbal Learning Test. *ISCED* International Standard Classification of Education. *MS* multiple sclerosis. *PPMS* primary progressive multiple sclerosis. *PRMS* progressive relapsing multiple sclerosis. *RRMS* relapsing-remitting multiple sclerosis. *SPMS* secondary-progressive multiple sclerosis. *SDMT* Symbol Digit Modalities Test.

Table S3. DMT treatment allocation.

DMT	Number of patients
Natalizumab	58
Ocrelizumab	15
Other DMTs[a]	12

Excludes patients who were lost to follow-up (three people with MS).

[a]Included teriflunomide, dimethyl fumarate, fingolimod, glatiramer acetate, azathioprine, methotrexate, cyclophosphamide.

DMT disease-modifying therapy.

Figure S1. Konectom™ app tests for cognitive and manual dexterity function.

A

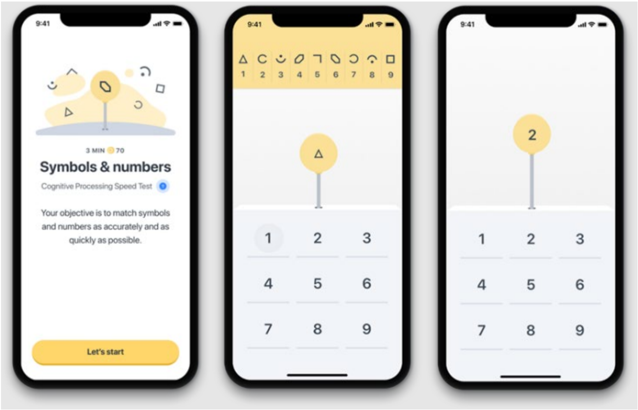

B

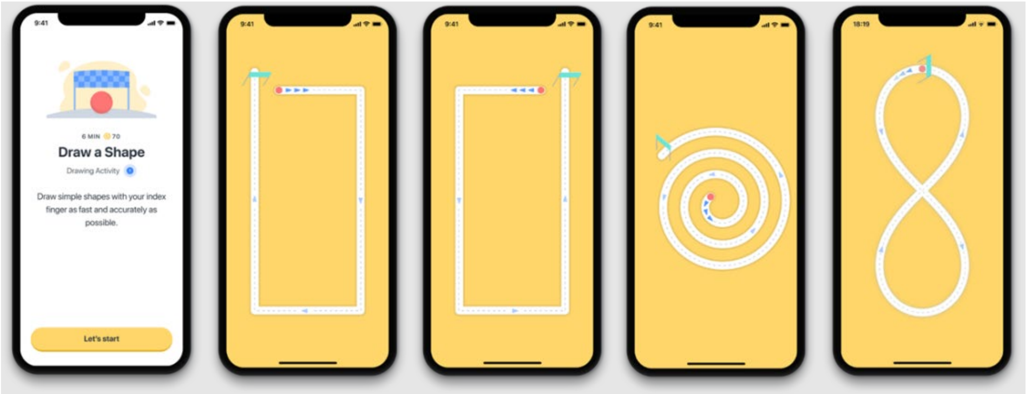

C

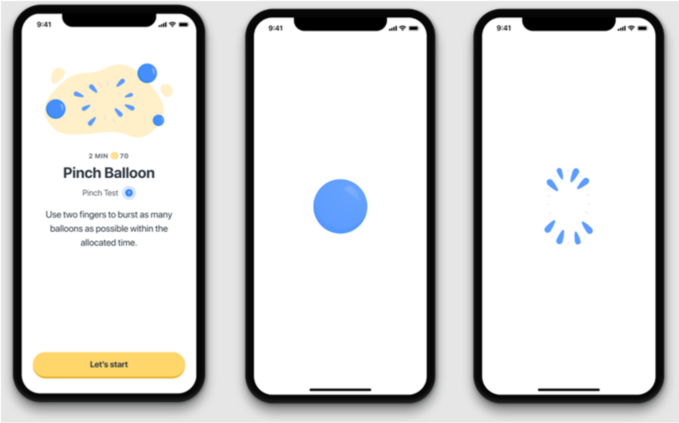

Konectom™ app interface: (**A**) Cognitive Processing Speed test; (**B**) Drawing test; (**C**) Pinching test.

Figure S2. Summary schedule of Konectom™ app tests for cognition, dexterity, and gait and posture.

Test Type	*Test name*	
**Experience Sampling**	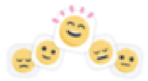	**Mood scale**
	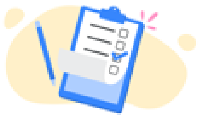	**MSIS-29**<br>Multiple Sclerosis Impact Scale
**Cognition**	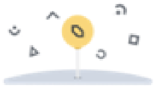	**Symbols & Numbers**<br>Cognitive Processing Speed
**Fine Motor**	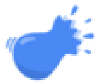	**Pinch Balloons**<br>Pinching Test
	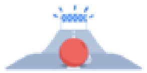	**Draw a Shape**<br>Drawing Test
**Gait & Posture**	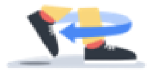	**Balance & U-turn**<br>Static Balance & U-Turn Test
	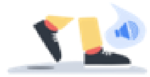	**Daily Walk**<br>6 Minute Walk Test

Figure S3. EDSS distribution.

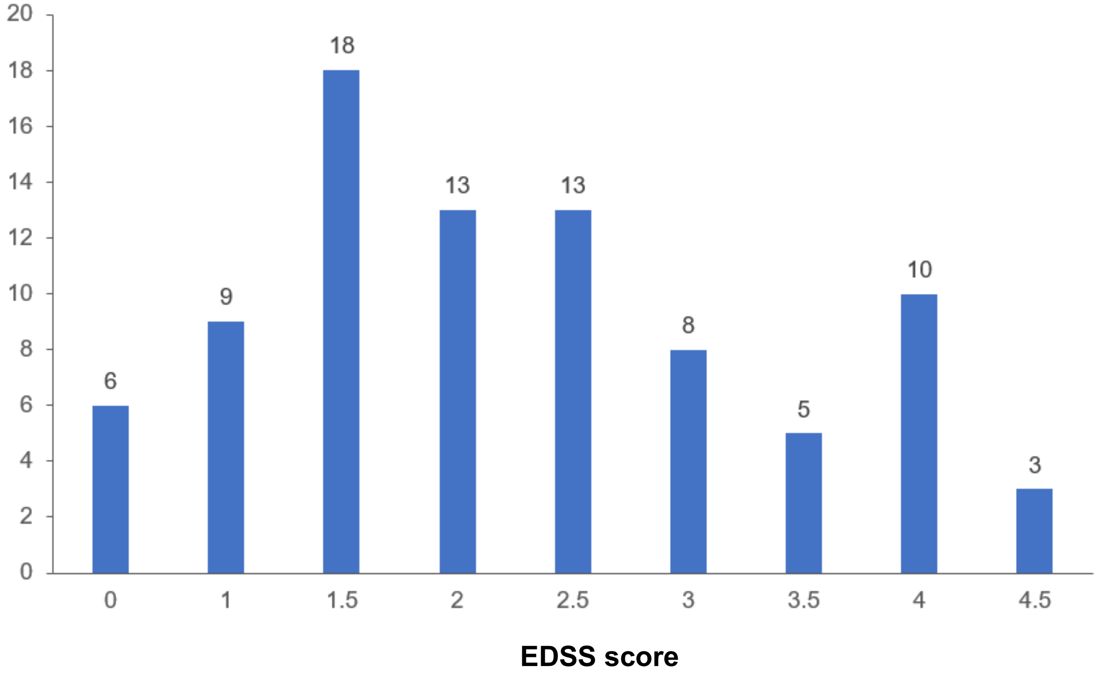

EDSS Expanded Disability Status Scale.

Figure S4. Process for selection and analysis of sensor-derived measures.

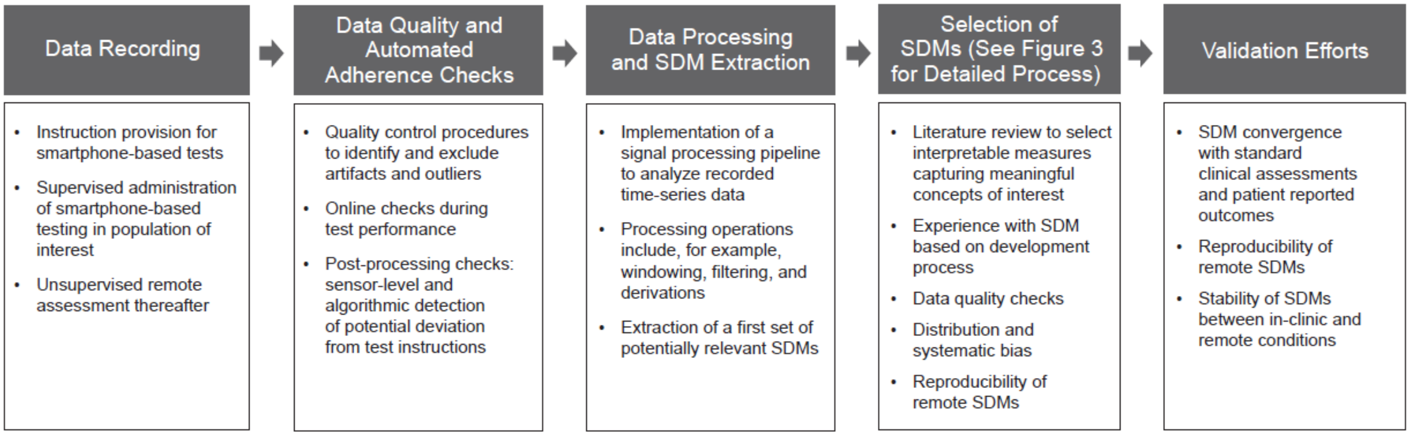

SDM sensor-derived measures.
